# Prognostic value of immune biomarkers in melanoma loco-regional metastases

**DOI:** 10.1371/journal.pone.0315284

**Published:** 2025-01-30

**Authors:** Emilia Hugdahl, Sura Aziz, Tor A. Klingen, Lars A. Akslen

**Affiliations:** 1 Department of Clinical Medicine, Centre for Cancer Biomarkers CCBIO, University of Bergen, Bergen, Norway; 2 Department of Pathology, Haukeland University Hospital, Bergen, Norway; 3 Department of Pathology, Vestfold Hospital, Tønsberg, Norway; The University of Queensland Faculty of Medicine, AUSTRALIA

## Abstract

The prognosis for patients with melanoma loco-regional metastases is very heterogenous. Adjuvant PD-L1-inhibitors have improved clinical outcome for this patient group, but the prognostic impact of tumour PD-L1 expression and number of tumour infiltrating lymphocytes (TILs) is still largely unknown. Here, we investigated the impact on survival for CD3, CD8, FOXP3 and PD-L1 TIL counts and tumour PD-L1 expression in melanoma loco-regional metastases. In a patient series of loco-regional metastases from nodular melanomas (n = 78; n = 26 skin metastases, n = 52 lymph node metastases), expression of PD-L1 in tumour cells and the number of CD3, CD8, FOXP3 and PD-L1 positive TILs were determined by immunohistochemistry on tissue microarray (TMA) slides. Due to limited tumour tissue in the paraffin blocks, 67 of the 78 cases were included for tissue microarrays. Low FOXP3 TIL count and negative tumour PD-L1 expression (cut off 1%) were both significantly associated with reduced survival in lymph node metastases. Low FOXP3 TIL count was significantly associated with low CD8, CD3 and PD-L1 TIL counts. Negative tumour PD-L1 expression was significantly associated with low CD8 and PD-L1 TIL count, large lymph node metastasis tumour size and presence of necrosis in lymph node metastases. Our findings demonstrate for the first time the negative prognostic value of low FOXP3 TIL count and confirm a negative prognostic value of negative tumour PD-L1 expression in melanoma lymph node metastases.

## Introduction

The prognosis for stage III melanoma is heterogenous, with 10-year survival rates varying from 24–88% [1]. Thus, there is a need for new prognostic biomarkers for better stratification of this patient group.

Melanoma is an immunogenic tumour, which implies a strong capacity to elicit adaptive immune responses. Still, the immunogenicity often does not result in elimination of tumour cells by the immune system. This paradoxical phenomenon is due to the remarkable capacity of melanoma to evade the host immune response [2].

Tumour infiltrating lymphocytes (TILs) are defined as lymphocytes that infiltrate tumour nests, in order to eliminate tumourigenic cells. Notably, the various TIL subsets have different functions in melanoma and can be identified by specific markers. CD3 is a pan T-cell marker, cytotoxic T-cells are identified by CD8, and helper T-cells are identified by CD4. FOXP3 is a marker of regulatory T-cells, and these lymphocytes suppress the effector lymphocytes and thereby regulate the tumour immune response [3].

Programmed cell death ligand 1 (PD-L1), expressed on both tumour cells and lymphocytes, and PD-1 receptor expressed on lymphocytes, both constitute so-called immune checkpoints, which inhibit an immune response against the tumour [4]. Since 2017, PD-L1 and PD-1 inhibitors have been used in the adjuvant setting of melanoma stage III disease, improving the clinical outcome. For stage IIIB and IIIC disease, 3-year recurrence-free survival is 65% and 54% respectively [5]. So, a relatively large proportion of stage III disease patients still do not benefit from immunotherapy. Unfortunately, PD-L1 expression has failed as a predictive marker of immunotherapy response in melanoma and is not used routinely [6].

In primary melanoma, like for most other cancer types, high numbers of CD8 positive and CD3 positive TILs are associated with a favourable prognosis [7–9]. High number of FOXP3 positive TILs have shown more diverse results but are most frequently associated with reduced survival [3,7,9]. Positive PD-L1 expression in tumour cells are most often associated with reduced survival [8,10–13].

For stage III melanoma, several studies have found significant association between improved survival and high numbers of CD3 positive and CD8 positive TILs [14–17]. Regarding FOXP3 TILs, only a few studies have been performed. One study found a trend for association with reduced survival for high numbers of FOXP3 positive TILs [14], while another study analyzing qPCR for FOXP3 found that patients with high levels of FOXP3 gene expression had significantly reduced survival [18]. High number of PD-L1 positive TILs was associated with reduced survival in one study of melanoma sentinel lymph node metastases [14]. Regarding PD-L1 expression in tumour cells, there is only one previous study analyzing its prognostic impact in melanoma lymph node metastases, which demonstrate reduced survival for PD-L1 negative tumours [19]. For stage IV melanoma, several studies have shown that tumours with negative PD-L1 expression have worse prognosis than tumours with positive expression [20–22], while others have found an opposite association with survival [23,24]. So, the prognostic value of PD-L1 expression in melanoma metastases is still unclear.

Here, we examined the prognostic impact of CD3, CD8, FOXP3 and PD-L1 TIL counts and of tumour PD-L1 expression in melanoma loco-regional metastases. A better understanding of the tumour immune microenvironment is imperative to improve stratification and ultimately treatment of stage III melanoma.

The population-based cohort and study design used in this study are the same as in our previously published work on prognostic value of angiogenic markers in melanoma [30]. In the present study, we also looked for associations between selected immune markers and angiogenesis characteristics without finding any. We therefore decided not to include these negative data.

## Material and methods

### Patients

This patient series consists of 255 consecutive cases of primary nodular cutaneous melanomas diagnosed at the Department of Pathology, Haukeland University Hospital (Bergen, Norway) during 1998–2008 [25,26]. During this time period, the sentinel node procedure was not performed in Norway, and our series therefore lacks complete staging. Clinical data and histologic variables of primary melanoma were included: sex, age at diagnosis (median age 70 years), tumour thickness according to Breslow [27] and microscopic tumour ulceration [28].

Complete information on patient survival, time and cause of death was available in all 255 cases. Last date of follow-up of survival, with information on cause of death from the Cancer Registry of Norway, was December 31, 2014. Median follow-up time for survivors was 115 months (range 72–203 months). During the follow-up period, 88 patients (35%) died of malignant melanoma and 76 (30%) died of other causes. Last date of follow-up of recurrent disease, including information from medical records, was February 29, 2016. Median follow-up time for survivors from time of first metastasis was 68 months (range 0–158 months). The data in this paper was accessed for research purposes on April 21, 2015. Since data for this paper was obtained from pathology records, the first author (EH) had access to information that could identify individual participants during data collection.

In addition, 78 paired biopsies from the first appearing local (skin; n = 26) or regional metastatic tumour (lymph nodes; n = 52) in this series were examined [25,29]. Clinical data for this subset of the cohort are listed in **S1 Table.** All cases were diagnosed before the use of immunotherapy was implemented. In cases with multiple metastases at the same time within the same organ compartment, we selected the largest tumour to represent the case. If the largest metastasis contained necrosis to the extent that representative tissue could not be sampled, then the largest tumour without such necrosis was chosen. Due to limited tumour tissue in the paraffin blocks, 67 of the 78 cases were available for tissue microarrays (n = 22 skin metastases, n = 45 lymph node metastases).

None of the cases with lymph node metastases had a synchronous skin metastasis, while six of the cases with skin metastases had a synchronous regional lymph node metastasis. When both lymph node metastasis and skin metastasis occurred at the same time, we selected the skin metastasis in this series because of their worse prognosis in the AJCC staging system. In skin metastases, the median tumour diameter was 8 mm (range 1–30), and necrosis was present in 8 cases (32%). In lymph node metastases, median tumour diameter was 30 mm (range 1–80), and necrosis was present in 33 cases (69%) [29]. Further, the median percentage of metastatic tumour replacement in each lymph node was 90% (range 30–100%).

The patient series used in this study lacks written consent, and this was approved by the Norwegian Data Inspectorate and the Regional Committee for Ethics in Research (Health Region III; 178.05) (REK 2009/564). All data were fully anonymized. The study was performed in accordance with the Declaration of Helsinki Principles.

### Clinico-pathologic variables

For all loco-regional metastases, clinical data and date of histologic diagnosis and site of first metastasis were registered. All slides of the loco-regional metastases were examined (EH, LAA) and the following histologic variables were recorded: maximum tumour diameter [29], tumour necrosis [29,30] and percentage of metastatic tumour replacement (lymph node metastases, SA).

### Tissue Microarray (TMA)

The TMA technique has been described and validated in several studies [31–33]. Three tissue cylinders (diameter 1.0 mm) from representative tumour areas identified on H&E stained slides, were punched from archival blocks and mounted into a recipient paraffin block using a custom made precision instrument (Minicore 3, Tissue Arrayer, Alphelys, France). Sections (5 μm) of the resulting TMA blocks were made by standard technique.

### Immunohistochemistry

Immunohistochemistry was performed on 5 μm standard tumour tissue sections of formalin-fixed paraffin embedded TMAs. All slides were dewaxed with xylene/ ethanol before antigen retrieval in a pressure cooker (Decloaking Chamber Plus, Biocare Medical, Concord, CA, USA), or in a microwave, in Target Retrieval Solution buffer pH 9 (S2367; DAKO/Agilent) for CD8, CD45, CD3 and FOXP3 and Target Retrieval Solution buffer pH 6 (S1699; DAKO/Agilent) for CD163. An endogenous block was applied prior to incubation with the primary antibodies to block for endogenous enzymatic activity. The slides were then incubated for 20 min-1 h with mouse monoclonal CD3, CD8, CD45, CD163 and FOXP3 antibodies (A0452 [Dako/Agilent, Santa Clara, CA, USA], M7103 [Dako], M0701 [Dako], NCL-L-CD163 [Dako], and M560044 [BD Biosciences, San Jose, CA, USA], respectively) followed by incubation with an appropriate HRP-En Vision (Dako), EnVision Mouse hrp for CD8; CD45, CD163 and FOXP3, EnVision rabbit HRP for CD3. Primary antibodies were omitted for the low controls. Low controls were obtained by omitting the primary antibody.

For the PD-L1, the staining was performed on the VENTANA BenchMark ULTRA IHC/ISH system (Ventana/Roche, Tucson, AZ), An antigen retrieval step using ULTRA Cell Conditioning Solution, CC1 (#950–224, Ventana/Roche, Tucson, AZ) was performed for 80 min, blocked with HRP-inhibitor (Inhibitor CM, part of Optiview DAB IHC detection kit) prior to a 16 min incubation at 36C with the prediluted SP263 PD-L1 antibody (#741–4905, Ventana/Roche, Tucson, AZ). Further detection was performed using OptiView HQ-kit (#760–700, Ventana/Roche, Tucson, AZ) for 8 + 8 min and stained with the OptiView DAB IHC Detection Kit (#760–700, Ventana/Roche, Tucson, AZ) for 8 min. The slides were thereafter stained with Hematoxilin II (#790–2208) and bluing reagent (#760–2037).

### Evaluation of PD-L1 positivity in tumour cells

Positive PD-L1 tumour staining was defined as ≥1% expression on tumour cells. Cases with positivity only in the cytoplasm were excluded. The percentage of PD-L1 tumour cell expression is given in each case. Areas with necrosis and heavy pigmentation were avoided. The evaluation was done three consecutive times by the same person (S.A.). and the highest value of the three evaluations was used in further analyses.

### Evaluation of FOXP3, CD8, CD3 and PD-L1 positive lymphocytes

Each case was stained with five markers: pan-lymphocytic marker (CD45), histiocytic marker (CD163), and other markers (CD3, CD8 and FOXP3). The first two markers were not counted, but were used either to identify lymphocytes (CD45) or histiocytes (CD163).

In this study, hot-spot areas of TILs were counted within the tumour tissue or in the intersection between tumour deposits and the lymphoid tissue, given that >50% tumour tissue (area) should be present within each region of interest (HPF, x400). In **S1 Fig**, the circle corresponds to one HPF (x400), whereas the rectangle within corresponds to the grid used.

The two hot-spots with the highest density of lymphocytes were selected. A special grid (area of 0.059 mm2) for the light microscopic (Zeiss) was then applied on the two most prominent hot-spots. The counted number of lymphocytes hence corresponds to an area of 0.059 mm2 x 2 = 0.118 mm2. The positive lymphocytes were counted in each case, both those crossing the lines, as well as the lymphocytes between the lines. The areas surrounding necrosis were avoided, and caution was considered when counting PD-L1 positive lymphocytes in the heavily pigmented cases. Areas with histiocytic infiltrates that showed cross reactivity with lymphocytic markers were avoided, after comparing such positivity with the morphology and with the concordant reactivity for the histiocytic marker CD163. The counting for each marker was done separately for each of the three TMA cores by the same author (S.A.), and the highest value of the three counts was used in further analyses. All analyses were performed using both the highest value of the three evaluations and the average value. All analyses gave very similar results when using average value or the highest value. We decided to use the highest value, as this represents the most immunologically active spot.

Raw data on TIL counts, PD-L1 tumour expression and clinical data is provided in **S2 Table.**

### Statistics

Statistical analyses were performed using the IBM Statistical package for the Social Sciences version 27 (IBM Corp. Armonk, NY). Association between different variables was assessed by the Spearman correlation test. Univariate analyses of time to death due to malignant melanoma were performed using the product-limit procedure (Kaplan-Meier method), and differences between categories were estimated by the log-rank test, with date of first metastasis as the starting point for analyses. Patients who died of other causes were censored at the date of death. All results were considered significant if p < 0.05.

In the statistical analyses, the cut-off points for categorization of continuous TIL counts and tumour PD-L1 expression were based on the quartiles, after considering the frequency distribution, the size of the subgroups and the number of events in each subgroup.

Spearman correlation test was used for analyses of inter-observer agreement of continuous variables. 25 randomly selected cases were assessed for PD-L1 positivity in tumour cells and CD8 and FOXP3 TIL counts by a second pathologist T.A.K. PD-L1 tumour positivity showed good inter-observer agreement (Spearman correlation coefficient 0.74, p< 0.05). Both CD8 and FOXP3 TIL counts showed good inter-observer agreement (Spearman correlation coefficient 0.98 and 0.91, respectively, p< 0.05 for both).

## Results

### TIL counts by expression of CD3, CD8, FOXP3, PD-L1, and tumour cell expression of PD-L1

Median counts of positive CD3, CD8, FOXP3 and PD-L1 TILs in lymph node and skin metastases are shown in **Table 1**.

**Table 1:** Median values with inter-quartile range of TIL counts in lymph node metastases and skin metastases (n = number of cases).

	PD-L1 TIL count	FOXP3 TIL count	CD8 TIL count	CD3 TIL count
Lymph node metastases (n = 45)	18 (0–95)	16 (4–38)	53 (20–133)	87.5 (35–303)
Skin metastases (n = 22)	0 (0–37)	8 (3–27)	21.5 (7–93)	49 (23–149)

The counted number of lymphocytes corresponds to an area of 0.118 mm2.

**Fig 1:**
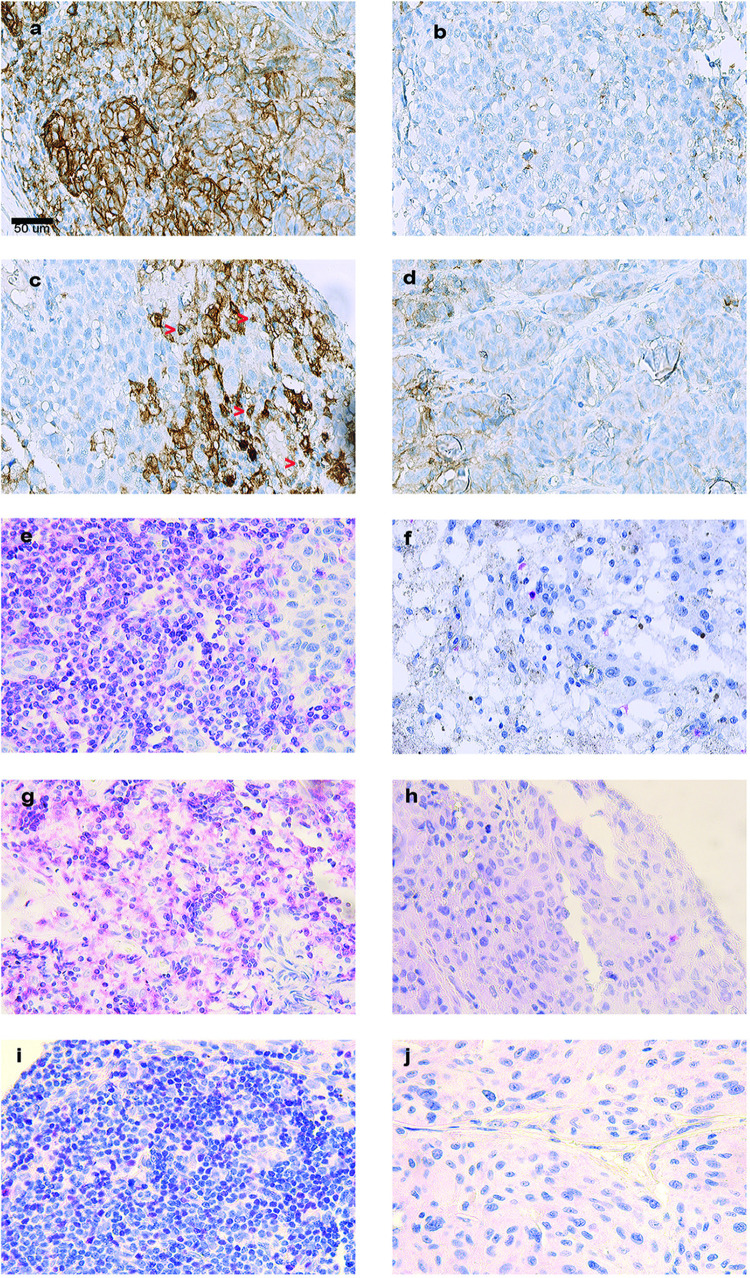
Immunohistochemical staining of melanoma lymph node metastases for PD-L1, FOXP3, CD8 and CD3. Examples of positive/negative tumour cell PD-L1 expression and high/low counts of the different TILs are shown. The magnification is the same in all pictures a-j (x400). The scalebar in **a** corresponds to 50um. **a:** Positive PD-L1 tumour expression. **b**: Negative PD-L1 tumour expression **c**: High PD-L1 lymphocyte count. **d**: Low PD-L1 lymphocyte count. **e**: High CD3 lymphocyte count. **f**: Low CD3 lymphocyte count. **g**: High CD8 lymphocyte count. **h**: Low CD8 lymphocyte count. **i**: High FOXP3 lymphocyte count. **j**: Low FOXP3 lymphocyte count.

In lymph node metastases, PD-L1 tumour expression was positive in 10 cases and negative in 34 cases. Median and mean percentage of PD-L1 tumour cell expression are shown in **Table 2**. For TIL counts, median values were used as cut-off, except for PD-L1 TIL count in skin metastases where the upper quartile was applied. For tumour cell PD-L1 expression, the upper quartile was also used, corresponding to a cut-off value of 1% tumour cell expression. **S3 Table** shows the values of PD-L1 tumour cell expression in cases with positive expression (≥ 1%) in lymph node metastases (n = 10). **Fig 1** shows melanoma lymph node metastases immunohistochemically stained with CD3, CD8, FOXP3 and PD-L1. An overview image of where the FOXP3 TILs were counted in relation to the metastatic deposits is shown in **S1 Fig**.

**Table 2:** PD-L1 tumour cell expression (%) in lymph node metastases and skin metastases.

	Median	Mean	Inter-quartile range	Minimum-maximum values
Lymph node metastases (n = 45)	0	5.0	0–1	0–100
Skin metastases (n = 22)	0	6.9	0–2	0–80

In lymph node metastases there was a significant positive correlation between FOXP3 TIL counts and CD8, CD3 and PD-L1 TIL counts (Spearman correlation coefficient 0.59, 0.70 and 0.61, respectively, p < 0.05 for all) (**Table 3**).

**Table 3:** Correlations between different TIL counts and PD-L1 tumour cell expression in lymph node metastases (n = 45).

	PD-L1 tumour cell expression (%)^a^	p^b^	PD-L1 TIL count (n)^a^	p^b^	FOXP3 TIL count (n)^a^	p^b^	CD8 TIL count (n)^a^	p^b^
**PD-L1 TIL count**	0.32	0.04						
**FOXP3 TIL count**	0.16	0.29	0.61	0.00				
**CD8 TIL count**	0.38	0.01	0.85	0.00	0.59	0.00		
**CD3 TIL count**	0.27	0.08	0.73	0.00	0.70	0.00	0.86	0.00

^a^Spearman correlation coefficient.

^b^Spearman correlation test.

PD-L1 tumour cell expression in lymph node metastases showed a significant positive correlation with CD8 and PD-L1 TIL counts (Spearman correlation coefficient 0.38 and 0.32, respectively, p < 0.05 for both) (**Table 3**), and a significant negative correlation with tumour necrosis and tumour diameter (Spearman correlation coefficient -0.33 and -0.31, respectively, p < 0.05 for both) (**Table 4**). CD8 and CD3 TIL counts showed significant negative correlations with percent tumour mass in lymph node metastases (**Table 4**).

**Table 4:** Correlation between PD-L1 tumour cell expression or various TIL counts and tumour necrosis or tumour size in lymph node metastases (n = 45).

	PD-L1 tumour cell expression (%) ^a^	p^b^	PD-L1 TIL count (n)^a^	p^b^	FOXP3 TIL count (n)^a^	p^b^	CD8 TIL count (n)	p^b^	CD3 TIL count (n)	p^b^
**Presence of tumour necrosis**	-0.33	0.03	-0.12	0.44	-0.28	0.06	-0.20	0.18	-0.24	0.12
**Tumour diameter (mm)**	-0.31	0.04	-0.08	0.60	-0.19	0.21	0.01	0.98	-0.17	0.76
**Percent tumour mass**	-0.18	0.25	-0.25	0.1	-0.25	0.1	-0.33	0.03	-0.35	0.02

^a^Spearman correlation coefficient.

^b^Spearman test.

The relationship between CD8 TIL counts and FOXP3 TIL counts, CD8 TIL counts and PD-L1 tumour expression and FOXP3 TIL count and PD-L1 tumour expression for each individual metastasis is shown in **S2 Fig**.

Hence, negative PD-L1 expression in lymph node metastases was significantly associated with low counts of CD8 and PD-L1 TILs and with presence of necrosis and large tumour diameter of lymph node metastasis. PD-L1 tumour cell expression in skin metastases showed no association with any subset of TILs, tumour diameter or tumour necrosis.

Among the six cases with synchronous skin and lymph node metastases, no obvious pattern of similarities or differences for CD8 or FOXP3 TIL counts was observed, while tumour cell PD-L1 expression was mostly negative in both skin and lymph nodes (S4 Table).

### Analysis of patient survival

**Fig 2:**
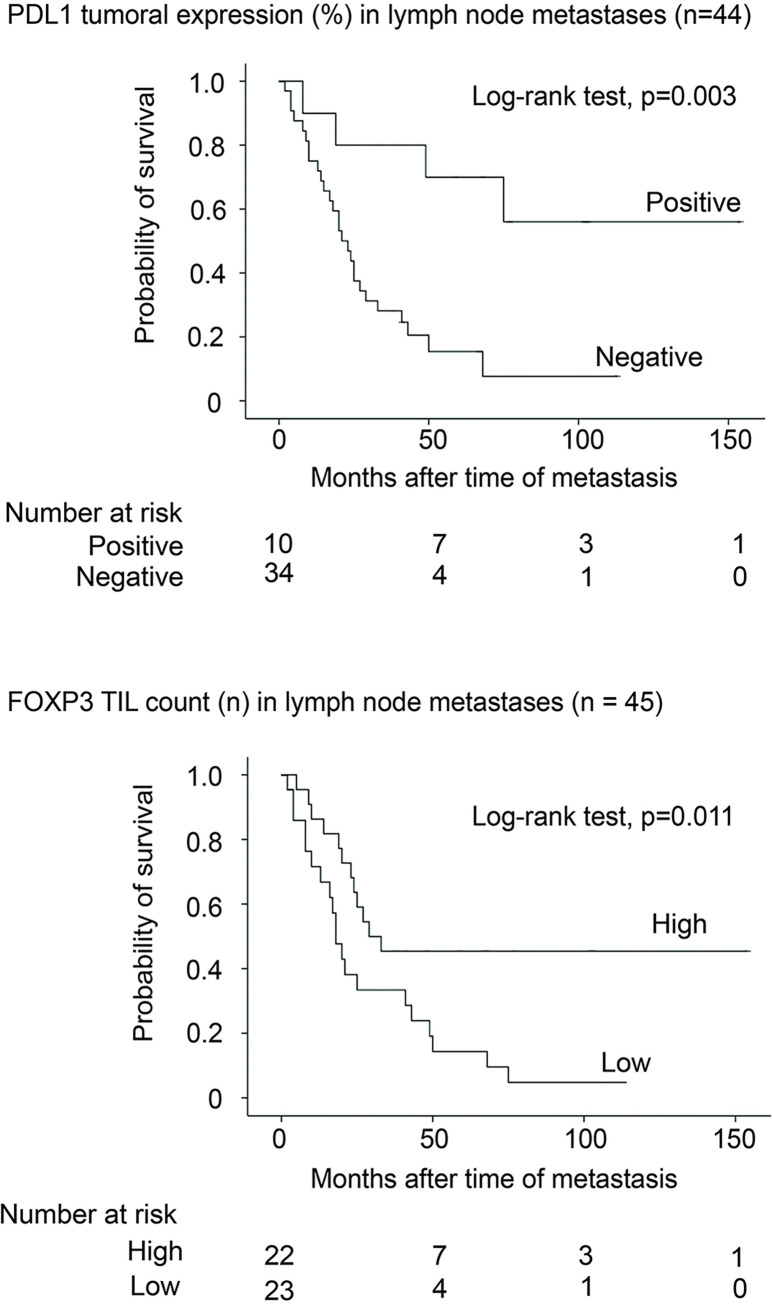
Survival by FOXP3 TIL count and PD-L1 tumour cell expression in lymph node metastases. Survival by FOXP3 TIL count was categorized according to the median (n = 16), and survival by PD-L1 tumour cell expression in melanoma lymph node metastases was categorized according to the upper quartile (1%). There was no association with survival in skin metastases (p = 0.65; log-rank test).

Low FOXP3 TIL count was associated with reduced survival in univariate analysis in lymph node metastases (p = 0.011; log-rank test) (**Fig 2**).

Negative tumour cell PD-L1 expression in lymph node metastases was significantly associated with reduced melanoma-specific survival in univariate analysis (p = 0.003; log-rank test) (**Fig 2**), whereas there was no association with survival in skin metastases (p = 0.64; log-rank test). PD-L1 TIL count was not associated with survival in lymph node (**S3 Fig**) or skin metastases in univariate analysis (p = 0.19 and 0.25; log-rank test).

CD8 TIL count showed no association with survival either in lymph node (**S3 Fig**) or skin metastases in univariate analysis (p = 0.31 and 0.55; log-rank test). Also, CD3 TIL counts showed no correlations with survival in lymph node (**S3** Fig) or skin metastases (p = 0.55 and 0.65; log-rank test) There was no prognostic correlation for CD8/FOXP3 ratio in skin or lymph node metastases (p = 0.28 and 0.46; log rank test).

## Discussion

As a novel finding, we demonstrate a prognostic impact of FOXP3 TIL count in a large patient series of melanoma lymph node metastases, with low FOXP3 TIL counts being associated with reduced survival. Further, this is the first study to show a prognostic value of both low FOXP3 TIL count and negative tumour cell PD-L1 expression in the same patient series.

Previously, a tendency for the opposite survival impact of FOXP3 TIL counts was shown in melanoma metastases by Kavakand et al. [34] and Knol et al. (FOXP3 gene expression by PCR) [18]. However, these findings cannot be directly compared to our data, as the studies were performed in the era of immunotherapy which might have influenced the survival impact. In primary melanoma, low FOXP3 TIL counts have shown improved survival [3,7,9]. Similar to our study, an association between reduced survival and low FOXP3 TIL count has been demonstrated for vulvar melanoma, head and neck, colorectal, and esophageal cancers, follicular lymphoma, diffuse large B-cell lymphoma, and Hodgkin lymphoma [35–37], although the underlying mechanism is yet to be understood. One potential explanation to this paradox, in which high numbers of the immunosuppressive FOXP3 TIL correlates with the best survival, may be a reflection of a compensatory mechanism of immune escape that follows CD8 TILs. Spranger et al. demonstrated that CD8 TIL positive tumours showed the highest expression of FOXP3 TILs, suggesting that the FOXP3 inhibitory effect is a negative feedback mechanism that follow, rather than precede, CD8 TIL infiltration [38]. We here suggest that it is the subset of melanoma metastases with lower CD8 TIL counts that showed the lowest values of the two immunosuppressive markers tumour cell PD-L1 expression and FOXP3 count. Thus, our observation supports the hypothesis by Spranger et al. that these inhibitory pathways might serve as negative feedback mechanisms that follow CD8 TIL infiltration.

Further, we found a low survival outcome associated with negative PD-L1 tumour cell expression in lymph node metastases. This support a similar observation in the only previous study of this by Madore et al. [39] in which negative tumour cell expression of PD-L1 was associated with reduced survival in melanoma lymph node metastases. However, the finding is not in line with the canonical understanding of the checkpoint mechanism of PD-L1 as inhibitor of the immune system, protecting tumour cells from immune reactions. According to this understanding, negative tumour PD-L1 expression should result in a favorable prognosis. Previous studies on primary melanoma have shown that negative PD-L1 expression in tumour cells are most often associated with improved survival [8,10–13], although one study observed the opposite in mucosal melanoma [40].

A possible explanation for our finding and the similar by Madore et al., may be that upregulation of PD-L1 tumour cell expression is the result of a strong immune response against melanoma mediated by CD8 positive TILs, as a negative feedback mechanism. Thus, PD-L1 is an indirect biomarker of antitumour immune response. This hypothesis was suggested by Taube et al. [21], who demonstrated that IFN-γ, a strong mediator of PD-L1 tumour cell expression, was detected at the interface of PD-L1 positive tumours and TILs, whereas none was found in PD-L1 negative tumours. IFN-γ is secreted by melanoma–specific CD8 TILs, and this cytokine was shown to rapidly induce PD-L1 in cultured melanomas. Consistent with the hypothesis that PD-L1 is a biomarker of anti-tumour immune response, Taube et al. demonstrated that the survival of patients with PD-L1 positive metastatic melanoma was significantly prolonged. Further support of this prognostic impact of tumour cell PD-L1 and of tumour PD-L1 being a marker of TIL antitumour response in melanoma, is given by Kluger et al. and Thierauf et al. [22,40]. The first study investigated different types of melanoma metastases, and the second study analyzed primary mucosal melanoma. Both studies confirm the association between positive tumour cell PD-L1 expression and high TIL count, and the association between positive tumour PD-L1 and improved survival. These findings are also in line with publications in other types of cancer, in which positive tumour PD-L1 expression was associated with a favorable outcome in non-small-cell lung cancer and breast carcinomas [41].

Hence, it is shown in several studies that melanoma with negative tumour cell PD-L1 expression lack immune infiltrates [21,22,40,42]. Madore et al. demonstrated an association of tumour cell PD-L1 negative melanoma lymph node metastases with low tumour mutation burden and a poor immune response gene expression profile [39].

Our study supports a significant association between negative tumour PD-L1 expression and low CD8 TIL count. A significant proportion of melanoma patients (40%) present with this combination of negative PD-L1 expression and scarce immune infiltrates [43]. These immunologically “cold” melanomas might have few TILs as a result of low neoantigen load (low tumour mutational burden). Possibly, such melanomas have strong simple genetic drivers creating few neoantigens [43]. Other explanations for tumours lacking immune infiltrates are defective antigen presentation or physical barriers to lymphocyte migration [44]. We demonstrate that negative tumour PD-L1 expression was significantly associated with presence of necrosis and large tumour diameter of lymph node metastasis, indicating that these are fast growing melanoma tumours. Thus, our study is the first to indicate a prognostic value of both low FOXP3 TIL count and negative tumour cell PD-L1 expression in the same patient series.

Median values of the different TIL counts were almost doubled in lymph node metastases compared to skin metastases. The same observation has been demonstrated previously in two studies [22,45]. Therefore, the prognostic value of the different TIL subsets was evaluated separately in the two groups of metastases. The distribution of tumour cell PD-L1 expression was quite similar in lymph node and skin metastases. However, tumour PD-L1 expression did not show prognostic impact for skin metastases, only in lymph node metastases. A possible explanation is that contrary to lymph node metastases, there was no association with CD8 TIL count in skin metastases. Thus, it seems that in our series of skin lesions, tumour PD-L1 expression is not a marker of TIL antitumour response. Among the six cases with synchronous skin and lymph node metastases, no obvious pattern of differences or similarities was found for TIL counts, while tumour cell PD-L1 expression was similar (mostly negative).

In the future, melanoma stratification based on the immune microenvironment will likely require next generation IHC, like mass cytometry, with spatial determination and quantification of multiple markers. This will allow for simultaneous evaluation of both immune cells, macrophages, stromal cells and cancer cell characterization, maintaining information of the morphology and structure of the tumour microenvironment. Ideally, further studies should also include information on mutational load, T-cell receptors and interferon-inflammatory signatures.

In conclusion, we report that patients with melanoma lymph node metastases with low FOXP3 TIL count and negative tumour cell PD-L1 expression have reduced survival, and that these metastases also have a low CD8 TIL count. Previously, it is known that such patients respond poorly to immunotherapy. How to improve the outcome for such patient groups with immunogenically “cold” metastases without any known immunosuppressive mechanisms turned on, is of utmost importance.

## Supporting information

S1 Table Patient characteristics (n = 67). (DOCX)

S2 Table Raw data for this series of 67 locoregional metastases. (XLSX)

S3 Table Cases (n = 10) with positive PD-L1 tumour cell expression (%) in lymph node metastases. (DOCX)

S4 Table Six cases with synchronous skin and lymph node metastases. (DOCX)

S1 Fig Overview image of where FOXP3 TILs were counted in relation to metastatic deposits. The circle indicates the HPF and the rectangle indicates the grid in which the TILs were counted.
(TIF)

S2 Fig Scatter plot showing the relationship between CD8 TIL counts and FOXP3 TIL counts, CD8 TIL counts and PD-L1 tumour expression, and FOXP3 TIL counts and PD-L1 tumour expression in lymph node metastases (n = 45). (TIF)

S3 Fig Survival by PD-L1, CD8 and CD3 counts in melanoma lymph node metastases. Survival by PD-L1, CD8 and CD3 TIL counts categorized according to the median (median values = 18, 53 and 87.5).
(TIF)

## References

[1] Gershenwald JE, Scolyer RA, Hess KR, Sondak VK, Long GV, Ross MI, et al. Melanoma staging: Evidence-based changes in the American Joint Committee on Cancer eighth edition cancer staging manual. CA: a cancer journal for clinicians. 2017;67(6):472–92. Epub 2017/10/14. doi: 10.3322/caac.21409 ; PubMed Central PMCID: PMC5978683. 29028110 PMC5978683

[2] Braeuer RR, Watson IR, Wu CJ, Mobley AK, Kamiya T, Shoshan E, et al. Why is melanoma so metastatic? Pigment Cell Melanoma Res. 2014;27(1):19–36. Epub 2013/10/11. doi: 10.1111/pcmr.12172 . 24106873

[3] Maibach F, Sadozai H, Seyed Jafari SM, Hunger RE, Schenk M. Tumor-Infiltrating Lymphocytes and Their Prognostic Value in Cutaneous Melanoma. Front Immunol. 2020;11:2105. Epub 2020/10/06. doi: 10.3389/fimmu.2020.02105 ; PubMed Central PMCID: PMC7511547. 33013886 PMC7511547

[4] McDermott DF, Atkins MB. PD-1 as a potential target in cancer therapy. Cancer Med. 2013;2(5):662–73. Epub 2014/01/10. doi: 10.1002/cam4.106 ; PubMed Central PMCID: PMC3892798. 24403232 PMC3892798

[5] Dimitriou F, Long GV, Menzies AM. Novel adjuvant options for cutaneous melanoma. Annals of oncology: official journal of the European Society for Medical Oncology / ESMO. 2021;32(7):854–65. Epub 2021/03/28. doi: 10.1016/j.annonc.2021.03.198 . 33771664

[6] Larkin J, Lao CD, Urba WJ, McDermott DF, Horak C, Jiang J, et al. Efficacy and Safety of Nivolumab in Patients With BRAF V600 Mutant and BRAF Wild-Type Advanced Melanoma: A Pooled Analysis of 4 Clinical Trials. JAMA Oncol. 2015;1(4):433–40. Epub 2015/07/17. doi: 10.1001/jamaoncol.2015.1184 . 26181250

[7] Fu Q, Chen N, Ge C, Li R, Li Z, Zeng B, et al. Prognostic value of -infiltrating lymphocytes in melanoma: a systematic review and meta-analysis. Oncoimmunology. 2019;8(7):1593806. Epub 2019/05/31. doi: 10.1080/2162402x.2019.1593806 ; PubMed Central PMCID: PMC6527267. 31143514 PMC6527267

[8] Chuang IC, Jang CS. Appraisal of clinicopathological prognosticators in advanced acral lentiginous melanoma with characterization of PD-L1 and CD8/CD4 immunoprofiles. Jpn J Clin Oncol. 2022. Epub 2022/06/07. doi: 10.1093/jjco/hyac093 . 35662346

[9] Sabbatino F, Scognamiglio G, Liguori L, Marra A, Anniciello AM, Polcaro G, et al. Perial Immune Infiltrate as a Prognostic Biomarker in Thin Melanoma. Front Immunol. 2020;11:561390. Epub 2020/10/30. doi: 10.3389/fimmu.2020.561390 ; PubMed Central PMCID: PMC7550791. 33117345 PMC7550791

[10] Lassalle S, Nahon-Esteve S, Frouin E, Boulagnon-Rombi C, Josselin N, Cassoux N, et al. PD-L1 Expression in 65 Conjunctival Melanomas and Its Association with Clinical Outcome. Int J Mol Sci. 2020;21(23). Epub 2020/12/04. doi: 10.3390/ijms21239147 ; PubMed Central PMCID: PMC7731195. 33266349 PMC7731195

[11] Bence C, Hofman V, Chamorey E, Long-Mira E, Lassalle S, Albertini AF, et al. Association of combined PD-L1 expression and tumour-infiltrating lymphocyte features with survival and treatment outcomes in patients with metastatic melanoma. J Eur Acad Dermatol Venereol. 2020;34(5):984–94. Epub 2019/10/19. doi: 10.1111/jdv.16016 . 31625630

[12] Yun S, Park Y, Moon S, Ahn S, Lee K, Park HJ, et al. Clinicopathological and prognostic significance of programmed death ligand 1 expression in Korean melanoma patients. J Cancer. 2019;10(13):3070–8. Epub 2019/07/10. doi: 10.7150/jca.30573 ; PubMed Central PMCID: PMC6590033. 31281485 PMC6590033

[13] Placke JM, Kimmig M, Griewank K, Herbst R, Terheyden P, Utikal J, et al. Correlation of PD-L1 expression in different tissue types and outcome of PD-1-based immunotherapy in metastatic melanoma—analysis of the DeCOG prospective multicenter cohort study ADOREG/TRIM. EBioMedicine. 2023;96:104774. Epub 2023/09/04. doi: 10.1016/j.ebiom.2023.104774 ; PubMed Central PMCID: PMC10483509. 37660535 PMC10483509

[14] Kakavand H, Vilain RE, Wilmott JS, Burke H, Yearley JH, Thompson JF, et al. PD-L1 expression, immune cell correlates and PD-1+ lymphocytes in sentinel lymph node melanoma metastases. Mod Pathol. 2015;28(12):1535–44. Epub 2015/09/26. doi: 10.1038/modpathol.2015.110 . 26403784

[15] Erdag G, Schaefer JT, Smolkin ME, Deacon DH, Shea SM, Dengel LT, et al. Immunotype and immunohistologic characteristics of tumor-infiltrating immune cells are associated with clinical outcome in metastatic melanoma. Cancer Res. 2012;72(5):1070–80. Epub 2012/01/24. doi: 10.1158/0008-5472.CAN-11-3218 ; PubMed Central PMCID: PMC3306813. 22266112 PMC3306813

[16] Falkenius J, Johansson H, Tuominen R, Frostvik Stolt M, Hansson J, Egyhazi Brage S. Presence of immune cells, low tumor proliferation and wild type BRAF mutation status is associated with a favourable clinical outcome in stage III cutaneous melanoma. BMC Cancer. 2017;17(1):584. Epub 2017/08/31. doi: 10.1186/s12885-017-3577-x ; PubMed Central PMCID: PMC5576332. 28851300 PMC5576332

[17] Bogunovic D O’Neill DW, Belitskaya-Levy I, Vacic V, Yu YL, Adams S, et al. Immune profile and mitotic index of metastatic melanoma lesions enhance clinical staging in predicting patient survival. Proc Natl Acad Sci U S A. 2009;106(48):20429–34. Epub 2009/11/17. doi: 10.1073/pnas.0905139106 ; PubMed Central PMCID: PMC2787158. 19915147 PMC2787158

[18] Knol AC, Nguyen JM, Quereux G, Brocard A, Khammari A, Dreno B. Prognostic value of tumor-infiltrating Foxp3+ T-cell subpopulations in metastatic melanoma. Exp Dermatol. 2011;20(5):430–4. Epub 2011/03/18. doi: 10.1111/j.1600-0625.2011.01260.x . 21410773

[19] Madore J, Vilain RE, Menzies AM, Kakavand H, Wilmott JS, Hyman J, et al. PD-L1 expression in melanoma shows marked heterogeneity within and between patients: implications for anti-PD-1/PD-L1 clinical trials. Pigment Cell Melanoma Res. 2015;28(3):245–53. Epub 2014/12/06. doi: 10.1111/pcmr.12340 . 25477049

[20] Larkin J, Chiarion-Sileni V, Gonzalez R, Grob JJ, Cowey CL, Lao CD, et al. Combined Nivolumab and Ipilimumab or Monotherapy in Untreated Melanoma. N Engl J Med. 2015;373(1):23–34. Epub 2015/06/02. doi: 10.1056/NEJMoa1504030 . 26027431 PMC5698905

[21] Taube JM, Anders RA, Young GD, Xu H, Sharma R, McMiller TL, et al. Colocalization of inflammatory response with B7-h1 expression in human melanocytic lesions supports an adaptive resistance mechanism of immune escape. Sci Transl Med. 2012;4(127):127ra37. Epub 2012/03/31. doi: 10.1126/scitranslmed.3003689 ; PubMed Central PMCID: PMC3568523. 22461641 PMC3568523

[22] Kluger HM, Zito CR, Barr ML, Baine MK, Chiang VL, Sznol M, et al. Characterization of PD-L1 Expression and Associated T-cell Infiltrates in Metastatic Melanoma Samples from Variable Anatomic Sites. Clin Cancer Res. 2015;21(13):3052–60. Epub 2015/03/20. doi: 10.1158/1078-0432.CCR-14-3073 ; PubMed Central PMCID: PMC4490112. 25788491 PMC4490112

[23] Massi D, Brusa D, Merelli B, Ciano M, Audrito V, Serra S, et al. PD-L1 marks a subset of melanomas with a shorter overall survival and distinct genetic and morphological characteristics. Annals of oncology: official journal of the European Society for Medical Oncology / ESMO. 2014;25(12):2433–42. Epub 2014/09/17. doi: 10.1093/annonc/mdu452 . 25223485

[24] Hino R, Kabashima K, Kato Y, Yagi H, Nakamura M, Honjo T, et al. Tumor cell expression of programmed cell death-1 ligand 1 is a prognostic factor for malignant melanoma. Cancer. 2010;116(7):1757–66. Epub 2010/02/10. doi: 10.1002/cncr.24899 . 20143437

[25] Hugdahl E, Kalvenes MB, Mannelqvist M, Ladstein RG, Akslen LA. Prognostic impact and concordance of TERT promoter mutation and protein expression in matched primary and metastatic cutaneous melanoma. British journal of cancer. 2017. Epub 2017/11/11. doi: 10.1038/bjc.2017.384 . 29123258 PMC5765228

[26] Hugdahl E, Kalvenes MB, Puntervoll HE, Ladstein RG, Akslen LA. BRAF-V600E expression in primary nodular melanoma is associated with aggressive tumour features and reduced survival. British journal of cancer. 2016;114(7):801–8. doi: 10.1038/bjc.2016.44 26924424 PMC4984864

[27] Breslow A. Thickness, cross-sectional areas and depth of invasion in the prognosis of cutaneous melanoma. Annals of surgery. 1970;172(5):902–8. Epub 1970/11/01. doi: 10.1097/00000658-197011000-00017 ; PubMed Central PMCID: PMC1397358. 5477666 PMC1397358

[28] Ladstein RG, Bachmann IM, Straume O, Akslen LA. Prognostic importance of the mitotic marker phosphohistone H3 in cutaneous nodular melanoma. The Journal of investigative dermatology. 2012;132(4):1247–52. Epub 2012/02/03. doi: 10.1038/jid.2011.464 . 22297638

[29] Hugdahl E, Bachmann IM, Schuster C, Ladstein RG, Akslen LA. Prognostic value of uPAR expression and angiogenesis in primary and metastatic melanoma. PLoS One. 2019;14(1):e0210399. Epub 2019/01/15. doi: 10.1371/journal.pone.0210399 ; PubMed Central PMCID: PMC6331131. 30640942 PMC6331131

[30] Ladstein RG, Bachmann IM, Straume O, Akslen LA. Tumor necrosis is a prognostic factor in thick cutaneous melanoma. Am J Surg Pathol. 2012;36(10):1477–82. Epub 2012/09/18. doi: 10.1097/PAS.0b013e31825a5b45 . 22982891

[31] Kononen J, Bubendorf L, Kallioniemi A, Barlund M, Schraml P, Leighton S, et al. Tissue microarrays for high-throughput molecular profiling of tumor specimens. Nat Med. 1998;4(7):844–7. Epub 1998/07/14. doi: 10.1038/nm0798-844 . 9662379

[32] Nocito A, Bubendorf L, Tinner EM, Suess K, Wagner U, Forster T, et al. Microarrays of bladder cancer tissue are highly representative of proliferation index and histological grade. J Pathol. 2001;194(3):349–57. Epub 2001/07/06. doi: 10.1002/1096-9896(200107)194:3&lt;349::AID-PATH887&gt;3.0.CO;2-D . 11439368

[33] Straume O, Akslen LA. Importance of vascular phenotype by basic fibroblast growth factor, and influence of the angiogenic factors basic fibroblast growth factor/fibroblast growth factor receptor-1 and ephrin-A1/EphA2 on melanoma progression. The American journal of pathology. 2002;160(3):1009–19. Epub 2002/03/14. doi: 10.1016/S0002-9440(10)64922-X ; PubMed Central PMCID: PMC1867162. 11891198 PMC1867162

[34] Kakavand H, Vilain RE, Wilmott JS, Burke H, Yearley JH, Thompson JF, et al. Tumor PD-L1 expression, immune cell correlates and PD-1+ lymphocytes in sentinel lymph node melanoma metastases. Mod Pathol. 2015. Epub 2015/09/26. doi: 10.1038/modpathol.2015.110 . 26403784

[35] Chłopik A, Selim MA, Peng Y, Wu CL, Tell-Marti G, Paral KM, et al. Prognostic role of tumoral PDL1 expression and peritumoral FoxP3+ lymphocytes in vulvar melanomas. Hum Pathol. 2018;73:176–83. Epub 2018/01/09. doi: 10.1016/j.humpath.2017.12.022 . 29307625

[36] Shang B, Liu Y, Jiang SJ, Liu Y. Prognostic value of tumor-infiltrating FoxP3+ regulatory T cells in cancers: a systematic review and meta-analysis. Sci Rep. 2015;5:15179. Epub 2015/10/16. doi: 10.1038/srep15179 ; PubMed Central PMCID: PMC4604472. 26462617 PMC4604472

[37] Tzankov A, Meier C, Hirschmann P, Went P, Pileri SA, Dirnhofer S. Correlation of high numbers of intratumoral FOXP3+ regulatory T cells with improved survival in germinal center-like diffuse large B-cell lymphoma, follicular lymphoma and classical Hodgkin’s lymphoma. Haematologica. 2008;93(2):193–200. Epub 2008/01/29. doi: 10.3324/haematol.11702 . 18223287

[38] Spranger S, Spaapen RM, Zha Y, Williams J, Meng Y, Ha TT, et al. Up-regulation of PD-L1, IDO, and T(regs) in the melanoma tumor microenvironment is driven by CD8(+) T cells. Sci Transl Med. 2013;5(200):200ra116. Epub 2013/08/30. doi: 10.1126/scitranslmed.3006504 ; PubMed Central PMCID: PMC4136707. 23986400 PMC4136707

[39] Madore J, Strbenac D, Vilain R, Menzies AM, Yang JY, Thompson JF, et al. PD-L1 Negative Status is Associated with Lower Mutation Burden, Differential Expression of Immune-Related Genes, and Worse Survival in Stage III Melanoma. Clin Cancer Res. 2016;22(15):3915–23. Epub 2016/03/11. doi: 10.1158/1078-0432.CCR-15-1714 . 26960397

[40] Thierauf J, Veit JA, Affolter A, Bergmann C, Grünow J, Laban S, et al. Identification and clinical relevance of PD-L1 expression in primary mucosal malignant melanoma of the head and neck. Melanoma research. 2015;25(6):503–9. Epub 2015/09/10. doi: 10.1097/CMR.0000000000000197 . 26352784

[41] Velcheti V, Schalper KA, Carvajal DE, Anagnostou VK, Syrigos KN, Sznol M, et al. Programmed death ligand-1 expression in non-small cell lung cancer. Lab Invest. 2014;94(1):107–16. Epub 2013/11/13. doi: 10.1038/labinvest.2013.130 . 24217091 PMC6125250

[42] Taube JM, Klein A, Brahmer JR, Xu H, Pan X, Kim JH, et al. Association of PD-1, PD-1 ligands, and other features of the tumor immune microenvironment with response to anti-PD-1 therapy. Clin Cancer Res. 2014;20(19):5064–74. Epub 2014/04/10. doi: 10.1158/1078-0432.CCR-13-3271 ; PubMed Central PMCID: PMC4185001. 24714771 PMC4185001

[43] Teng MW, Ngiow SF, Ribas A, Smyth MJ. Classifying Cancers Based on T-cell Infiltration and PD-L1. Cancer Res. 2015;75(11):2139–45. Epub 2015/05/16. doi: 10.1158/0008-5472.CAN-15-0255 ; PubMed Central PMCID: PMC4452411. 25977340 PMC4452411

[44] Trujillo JA, Sweis RF, Bao R, Luke JJ. T Cell-Inflamed versus Non-T Cell-Inflamed Tumors: A Conceptual Framework for Cancer Immunotherapy Drug Development and Combination Therapy Selection. Cancer immunology research. 2018;6(9):990–1000. Epub 2018/09/06. doi: 10.1158/2326-6066.CIR-18-0277 ; PubMed Central PMCID: PMC6145135. 30181337 PMC6145135

[45] Balatoni T, Mohos A, Papp E, Sebestyén T, Liszkay G, Oláh J, et al. Tumor-infiltrating immune cells as potential biomarkers predicting response to treatment and survival in patients with metastatic melanoma receiving ipilimumab therapy. Cancer immunology, immunotherapy: CII. 2018;67(1):141–51. Epub 2017/10/11. doi: 10.1007/s00262-017-2072-1 . 28988380 PMC11028067

