# Supplementary material for: Prognostic value of immune biomarkers in melanoma loco-regional metastases
Source: PLoS One. 2025 Jan 30;20(1):e0315284. doi: 10.1371/journal.pone.0315284 (PMC11781691; doi:10.1371/journal.pone.0315284)
Supplement: S1 Table — (DOCX) [file pone.0315284.s001.docx]

**Table S1. Patient characteristics (n = 67).**

| Age (years)  Median (range) | 62 (21-87) |
| --- | --- |
| Sex  Men  Women | 39 (58 %)  28 (42 %) |
| Tumor anatomic site  Head/neck  Truncus  Upper extremities  Lower extremities  Other sites | 11 (16 %)  24 (36 %)  16 (24 %)  15 (22 %)  1 (2 %) |
| Status at last follow-up  Alive  Death from melanoma  Death from other causes | 12 (18 %)  49 (73 %)  6 (9 %) |
