# Supplementary material for: Prognostic value of immune biomarkers in melanoma loco-regional metastases
Source: PLoS One. 2025 Jan 30;20(1):e0315284. doi: 10.1371/journal.pone.0315284 (PMC11781691; doi:10.1371/journal.pone.0315284)
Supplement: S3 Table — (DOCX) [file pone.0315284.s003.docx]

| **Table S3. Cases (n=10) with positive PD-L1 tumour cell expression (%) in lymph node metastases** | |
| --- | --- |
|  |  |
| Case 585 | 1 |
| Case 642 | 100 |
| Case 684 | 1 |
| Case 736 | 1 |
| Case 758 | 1 |
| Case 793 | 10 |
| Case 794 | 5 |
| Case 804 | 1 |
| Case 805 | 20 |
| Case 866 | 80 |
