# Supplementary material for: Prognostic value of immune biomarkers in melanoma loco-regional metastases
Source: PLoS One. 2025 Jan 30;20(1):e0315284. doi: 10.1371/journal.pone.0315284 (PMC11781691; doi:10.1371/journal.pone.0315284)

| **Table S4. Six cases with synchronous skin and lymph node metastases** | | | | | | |
| --- | --- | --- | --- | --- | --- | --- |
|  | CD8 TIL count | p | FOXP3 TIL count | p | PD-L1 tumour expression | p |
| Spearman correlation coeffisient | 0.37 | ns | 0.58 | ns | - | - |
|  |  |  |  |  |  |  |
| Case nr 576 |  |  |  |  |  |  |
| skin | 22 |  | 18 |  | 1 |  |
| lymph node | 42 |  | 8 |  | 0 |  |
| Case nr 645 |  |  |  |  |  |  |
| skin | 3 |  | 0 |  | 0 |  |
| Lymph node | 0 |  | 0 |  | 0 |  |
| Case nr 704 |  |  |  |  |  |  |
| skin | 100 |  | 17 |  | 0 |  |
| Lymph node | 12 |  | 27 |  | 0 |  |
| Case nr 718 |  |  |  |  |  |  |
| skin | 32 |  | 20 |  | 0 |  |
| Lymph node | 31 |  | 8 |  | 0 |  |
| Case nr 822 |  |  |  |  |  |  |
| skin | 21 |  | 5 |  | 0 |  |
| Lymph node | 19 |  | 8 |  | 0 |  |
| Case nr 886 |  |  |  |  |  |  |
| skin | 43 |  | 10 |  | 0 |  |
| Lymph node | 55 |  | 5 |  | 0 |  |


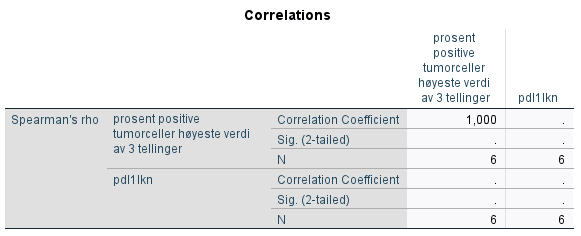

Supplement: S4 Table — (DOCX) [file pone.0315284.s004.docx]
