# Supplementary figures and images for: Prognostic value of immune biomarkers in melanoma loco-regional metastases
Source: PLoS One. 2025 Jan 30;20(1):e0315284. doi: 10.1371/journal.pone.0315284 (PMC11781691; doi:10.1371/journal.pone.0315284)

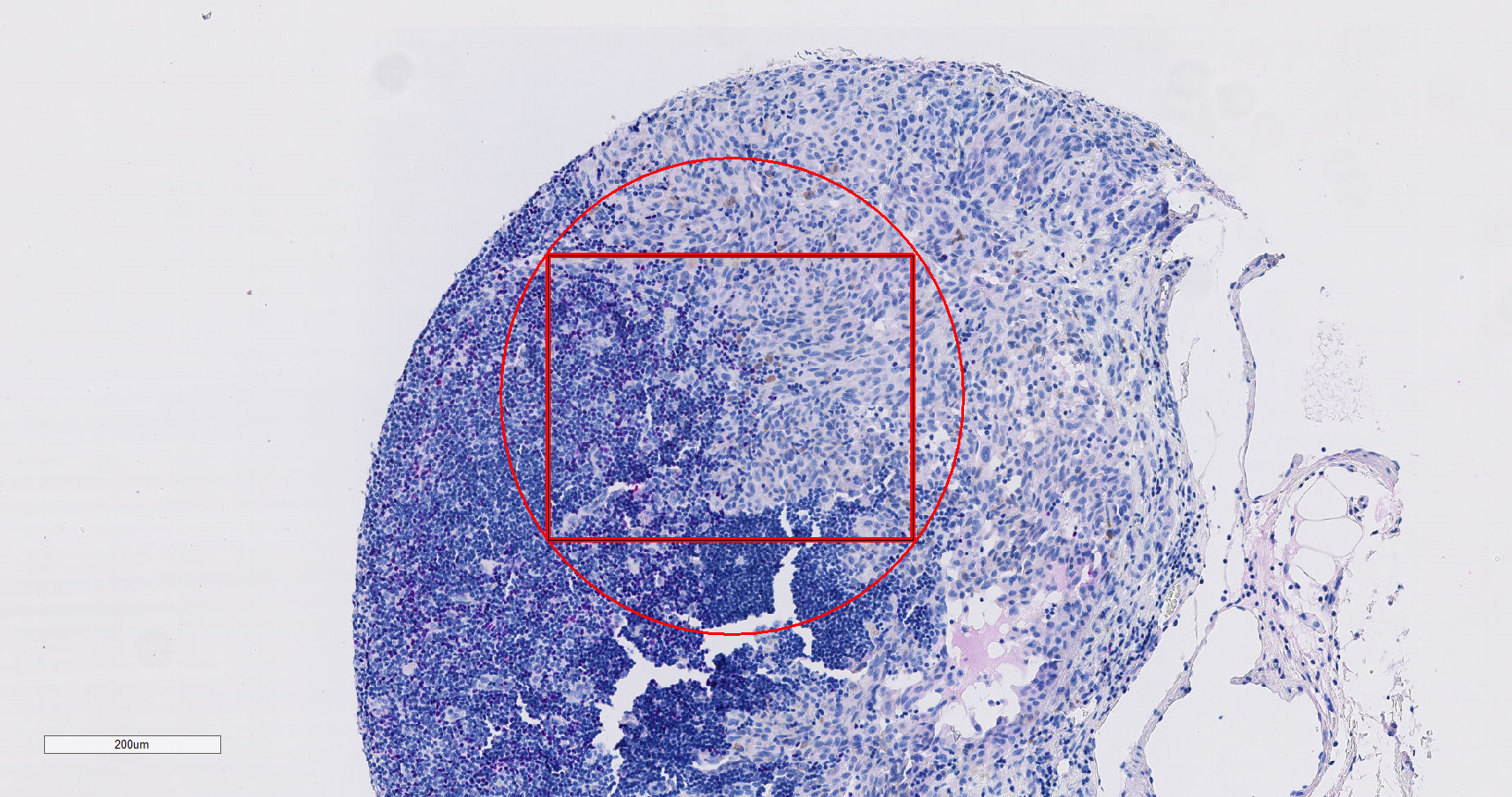

Supplement: S1 Fig — The circle indicates the HPF and the rectangle indicates the grid in which the TILs were counted. (TIF) [file pone.0315284.s005.tif]

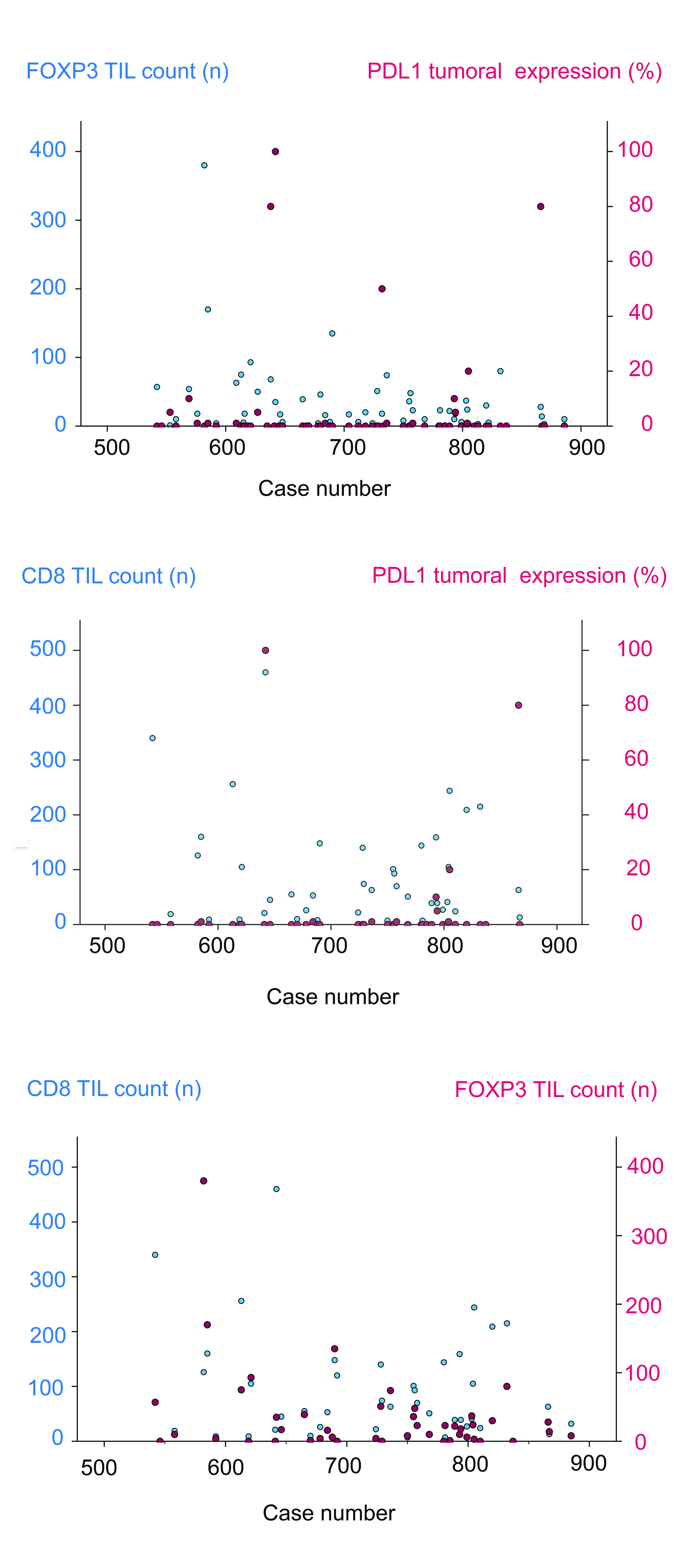

Supplement: S2 Fig — (TIF) [file pone.0315284.s006.tif]

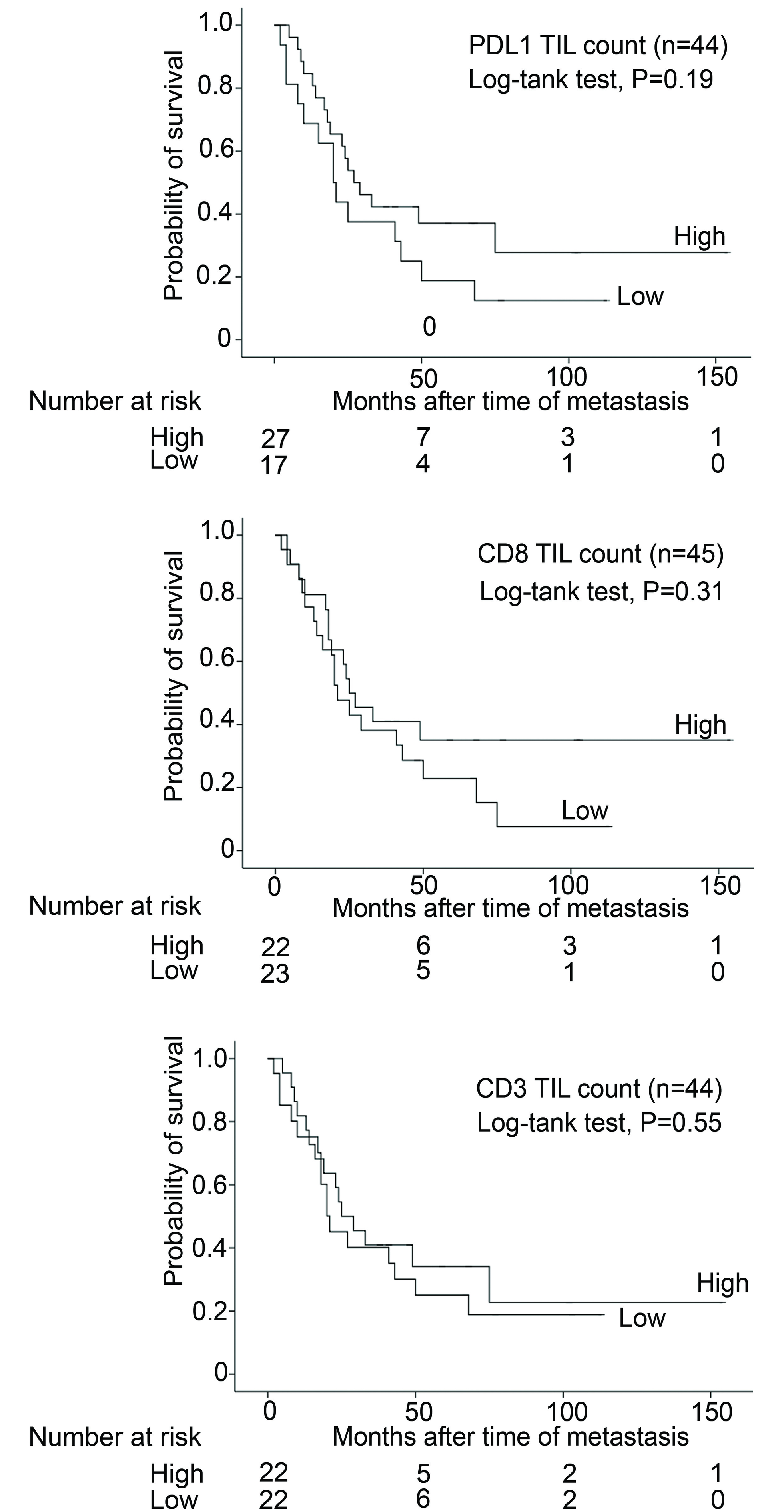

Supplement: S3 Fig — Survival by PD-L1, CD8 and CD3 TIL counts categorized according to the median (median values = 18, 53 and 87.5). (TIF) [file pone.0315284.s007.tif]
